# Dynamics of soil microbiome throughout the cultivation life cycle of morel (*Morchella sextelata*)

**DOI:** 10.3389/fmicb.2023.979835

**Published:** 2023-02-22

**Authors:** Chen Zhang, Xiaofei Shi, Jiexiong Zhang, Yesheng Zhang, Wen Wang

**Affiliations:** ^1^School of Ecology and Environment, Northwestern Polytechnical University, Xi’an, China; ^2^The Germplasm Bank of Wild Species, Yunnan Key Laboratory for Fungal Diversity and Green Development, Kunming Institute of Botany, Chinese Academy of Sciences, Kunming, China; ^3^Shandong Junsheng Biotechnologies Co., Ltd., Liaocheng, China

**Keywords:** metagenomics, microbial community, morel (*Morchella sextelata*), primordium formation, fruiting body maturation

## Abstract

Although *Morchella sextelata* (morel) is a well-known, edible, and medicinal fungus widely cultivated in China, the dynamics and roles of its soil microbiome during cultivation are unclear. Using rhizosphere soil samples collected throughout the *M. sextelata* cultivation life cycle, we conducted a high-throughput metagenomic sequencing analysis, with an emphasis on variations in soil microbial composition, characteristic biomarkers, and ecological functions. We found that microbial relative abundance, alpha diversity, and structure varied significantly among fungal growth stages. A total of 47 stage-associated biomarkers were identified through a linear discriminant analysis of effect size. In addition, horizontal comparison of soil microbiomes exhibiting successful and failed primordium formation further confirmed primordium-associated microbes with possible key roles in primordium formation. A microbial function analysis revealed that nutrient metabolism-related pathways were enriched during mycelium and fruiting body stages, whereas the signal transduction pathway was enriched during the primordium stage. This result indicates that diverse microbes are required at different growth stages of *M. sextelata*. Our research has revealed the dynamic scenario of the soil microbiome throughout the cultivation life cycle of *M. sextelata*. The high-resolution microbial profiles uncovered in the present study provide novel insights that should contribute to the improvement of morel cultivation using microbial inoculants.

## 1. Introduction

Morels (*Morchella* spp.) are well-known, edible, and medicinal fungi with unique, honeycomb-like fertile tissues (Pilz et al., 2007) and an abundance of amino acids, polysaccharides, and trace elements (Tietel and Masaphy, 2018). According to various studies, morels are beneficial to human health, with significant tumor-inhibitory, immune-enhancing, and antioxidant and antibacterial properties (Heleno et al., 2013; Wu et al., 2020; Sunil and Xu, 2022).

Morel cultivation has been a research focus worldwide, with an early indoor cultivation of *M. rufobrunnea* in the 1980s in the United States (Ower, 1982). In recent decades, the cultivation area of several black morels (*M. sextelata, M. importuna,* and *M. exima*) in China has rapidly expanded to 10,000 ha, with an average yield more than 3,000 kg/ha (Tang et al., 2021; Zhao et al., 2021). The cultivation process includes sowing of cultured spawn in soil, coverage with exogenous nutrients for approximately 10–15 days (until the soil surface is covered with mycelia), and management of fruiting bodies under appropriate growth conditions (Li et al., 2016). In a recent study of *M. importuna*, the carbon used to support fruiting body growth was initially transferred from exogenous nutrient bags to the soil, whereas required nitrogen seemed to originate directly from soil (Tan et al., 2019). Although large-scale cultivation of morels has been achieved in China, several major obstacles still exist, including yield instability (Du and Yang, 2021), susceptibility to pathogen infection (Guo et al., 2016; He et al., 2017, 2018), and environmental fluctuations. To address these challenges and improve commercial morel cultivation in the field, an understanding of the soil microbiome during the cultivation life cycle of morels would be useful.

Soil microbiome structure and function affect plant development (Edwards et al., 2015; Chen et al., 2019; Xiong et al., 2021). Numerous studies have clarified mechanisms of plant growth-promoting rhizobacteria (Nerva et al., 2022; Singh R. K. et al., 2022; Upadhyay et al., 2022) and highlighted the great potential of microbial inoculants (Deng et al., 2022; Singh P. et al., 2022; Upadhyay and Chauhan, 2022). Soil microbes also have important functions in the growth of mushrooms, such as *Agaricus bisporus* (Wang et al., 2016), *Ganoderma lucidum* (Zhang et al., 2018), *Phlebopus portentosus* (Yang et al., 2019), and *Stropharia rugosoannulata* (Gong et al., 2018). Possible key functions of the soil microbiome during mushroom growth are the following: (i) conversion of lignocellulosic feedstock into a selective, nutrient-rich fertilizer for mushroom growth; (ii) interaction with the mushroom itself during mycelial elongation and proliferation stages; and (iii) induction of primordium and ascocarp formation during cultivation (Kertesz and Thai, 2018). In contrast, some bacterial and fungal taxa in soil substrates can act as pathogens of cultivated mushrooms, leading to reduced yield and severe quality loss.

The nutritional types of *Morchella* vary from soil-saprophytic to symbiotic (Larson et al., 2016; Longley et al., 2019). The three main cultivated species (*M. sextelata*, *M. importuna,* and *M. exima*) are soil-saprophytic, which must be covered with soil during cultivation while the mycelium acquires nutrients from soil and surrounding substrate. Longley et al. (2019) analyzed the composition of soil bacteria and fungi associated with *M. rufobrunnea* at primordium and fruiting body stages by 16S rRNA and ITS amplicon sequencing and found that *Gilmaniella* and *Bacillus* were predominant in substrates. In addition, Benucci et al. (2019) examined the microbial community composition of *M. sextelata* ascocarps and soils beneath the ascocarps by 16S rRNA and ITS amplicon sequencing, and Orlofsky et al. (2021) studied the composition and function of the soil microbiome beneath young and mature fruiting bodies of *M. rufobrunnea* in the field. Furthermore, Tan et al. (2021) comparatively analyzed soil microbial composition, nutrient transformation, and key enzyme activities after inoculation of *M. importuna* into semi-synthetic substrates and proposed a conceptual illustration of the ecophysiological factors influencing morel fructification. Despite these advances in elucidating the effect of the soil microbiome, a comprehensive understanding of soil microbiome dynamics from seeding to harvesting, i.e., during mycelium elongation, primordium formation, and ascocarp maturation, is lacking, as current 16S rRNA and ITS amplicon sequencing methods cannot easily identify functional genes of the soil microbiome in detail. A life cycle investigation of the soil microbiome across all morel growth stages is crucial to: (1) determine how the soil microbiome dynamically varies with growth stages during morel cultivation, (2) identify key biomarkers associated with variations shaping the soil microbiome, (3) understand the ecological functions of these soil microbes, and (4) reveal how these microbes affect morel growth and development.

In this study, we hypothesized that the soil microbiome changes significantly along with morel growth, especially the primordium and fruiting body stages. We also hypothesized that microbiota drive changes in soil ecological functions associated with morel growth and development. To test these hypotheses, we analyzed the dynamic succession of the soil microbiome and identified stage-associated characteristic biomarkers from sowing to harvesting in *M. sextelata* during mycelium (days 1, 10, and 23), primordium (day 40), and fruiting body (days 55, 65, and 90) stages. We also compared functional pathways of the soil microbiome at different growth stages to understand the ecological functions and potential mechanisms of microbes promoting morel growth and development.

## 2. Materials and methods

### 2.1. Morel cultivation and soil sampling

**Figure 1:**
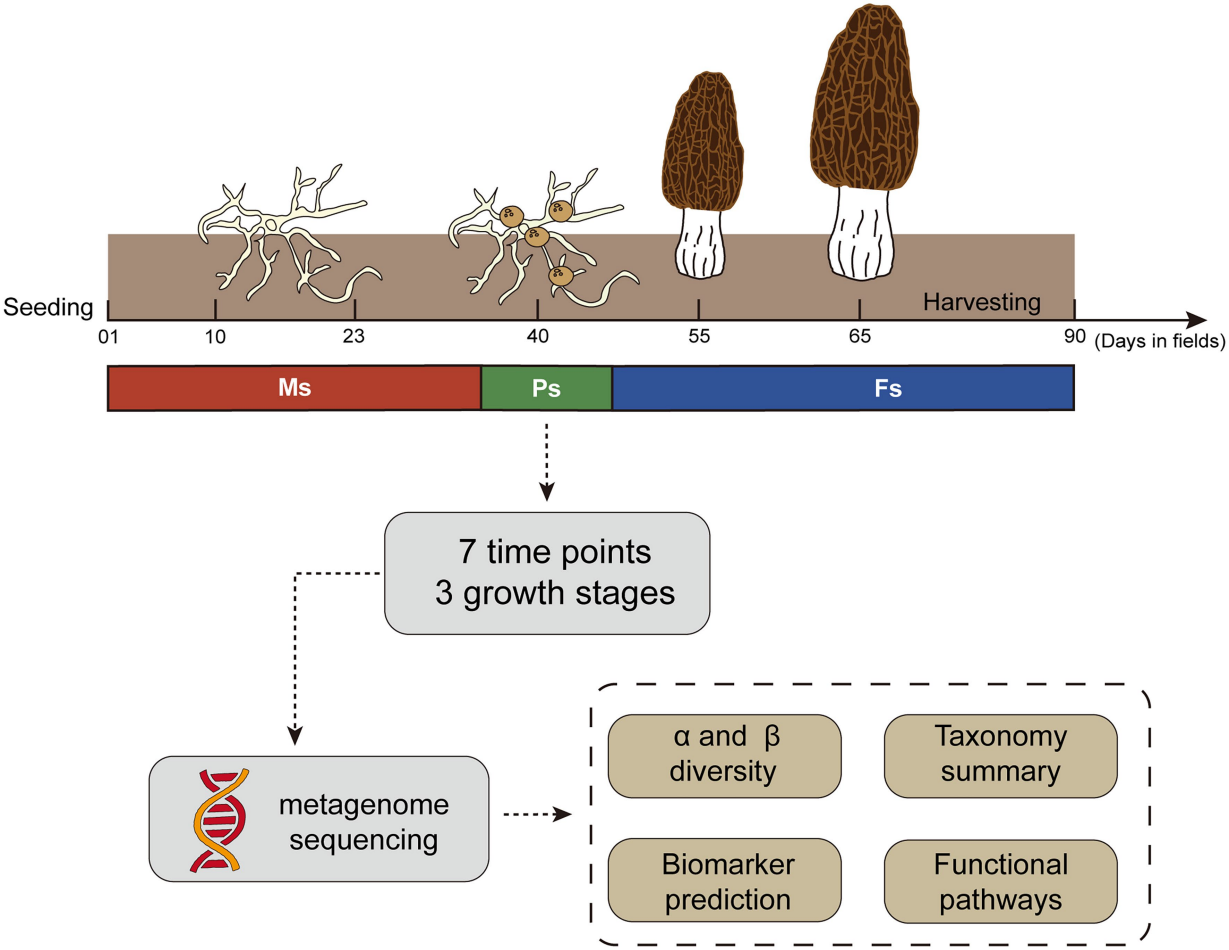
Outline of the study design and sampling strategy used throughout the *Morchella sextelata* cultivation life cycle. Ms, mycelium stage; Ps, primordium stage; Fs, fruiting body stage.

Morels (*M. sextelata*) were cultivated in a plastic greenhouse in Jinji, Longyang County (25.16° N, 99.24° E; 1,600 m above sea level), Yunnan, China, in the Winter of 2020. The cultivation procedure followed a standard technical protocol (Liu et al., 2017). During the key growth stages of *M. sextelata*, representative rhizosphere soil samples were collected around *M. sextelata* mycelium (days 01, 10, and 23), primordium (day 40), and fruiting body (days 55, 65, and 90; Figure 1; Supplementary Table S1). After removal of the top 2 cm of soil, rhizosphere soil at a depth of 2–8 cm was collected with a 10-cm soil auger from three sites located 2–3 m apart on a ridge, with the three samples then thoroughly mixed as one composite sample. At each sampling period, we collected three composite samples as replicates from three different ridges. A total of 21 composite samples were collected at seven time points. We also collected five composite soil samples between days 55 and 65 with failed primordium formation from a nearby, separate greenhouse (distance < 500 m) in which morels were sown at approximately the same time (Supplementary Table S1) and cultivated similarly (Liu et al., 2017). All samples were rapidly frozen with liquid nitrogen and stored at −80°C for subsequent DNA extraction.

### 2.2. DNA extraction and shotgun metagenomic sequencing

DNA extraction and shotgun metagenomic sequencing were performed by Novogene (Beijing, China). Total DNA was extracted from samples using a PowerSoil DNA extraction kit (MO BIO, Carlsbad, CA, United States) and then qualified and quantified by agarose gel electrophoresis and nano-spectrophotometry. Qualified DNA was used for construction of a metagenomic sequencing library, which was analyzed on the Illumina NovaSeq 6000 platform (Illumina, San Diego, CA, United States) with a sequencing read length of 150 bp and an insert fragment size of 350 bp. All sequence data were deposited at NCBI under BioProject accession number PRJNA841746.

### 2.3. Bioinformatic analyses

After removal of adapter sequences and trimming of low-quality sequences based on a 5-bp sliding window (quality < 20, read accuracy < 99%), sequences longer than 50 bp were retained for further analysis (Bolger et al., 2014). The sequencing data were aligned to the reference genome of *M. sextelata* using the Kneaddata v0.7.4 pipeline with Bowtie2 (Langmead and Salzberg, 2012), and the sequences from *M. sextelata* genome were then removed. The processed data were assembled with Megahit v1.2.9 using the deBruijn graph method (Li et al., 2015). The metagenome assembly results were evaluated in Quast v5.0.2 (Gurevich et al., 2013). Coding sequences were predicted with Prodigal v2.6.3 and further clustered using CD-HIT v4.8.1, with 95% amino acid identity used as a criterion, to obtain a non-redundant gene catalog (Hyatt et al., 2010; Fu et al., 2012). Gene abundance was calculated in Salmon v1.4.0 based on coverage of metagenomic gene regions (Patro et al., 2017). Accurate *k-mer* matching against the NCBI-NT database was performed with Kraken2 v2.1.1, and annotated microbial taxonomic profiles were generated by the lowest common ancestor method (Wood et al., 2019). Non-redundant gene sequences were compared against the eggNOG v5.0 database using Diamond v0.9.36 for annotation of microbial functional profiles (Buchfink et al., 2015; Huerta-Cepas et al., 2019). Finally, soil microbiome taxonomic and functional composition tables were obtained for further statistical analyses and visualization.

### 2.4. Statistical analyses and visualization

Prior to analysis, data were quality filtered to remove microbial taxa with a relative abundance < 0.01%. Rarefaction curves were used to assess data richness from the results of sampling. The top 10 phyla at different time points were visualized using the histogram function in the R package ggplot2 (Ginestet, 2011). Alpha diversity based on Shannon’s and Simpson’s indexes was evaluated using the R package vegan (Dixon, 2003). Microbiome structure (beta diversity) was calculated based on Bray–Curtis distances (Ricotta and Podani, 2017) and visualized by principal coordinate analysis (PCoA) and non-metric multidimensional scaling (NMDS). A linear discriminant analysis of effect size (LEFSe) with *p*-value < 0.05 and LDA score > 2 thresholds was conducted to identify characteristic biomarkers at different *M. sextelata* growth stages (Segata et al., 2011). Biomarkers were sorted by LDA score from largest to smallest, and the R package pheatmap was used to display the average relative abundance of biomarkers at each growth stage. STAMP v2.1.3 was used to examine the abundance of functional pathways between different growth stages (Parks et al., 2014), and significant differences among samples were assessed with a *t*-test. The QIIME2 v2021.11.0 pipeline and R v4.1.1 were used throughout the study for data processing, analysis, and visualization (Bolyen et al., 2019).

## 3. Results

### 3.1. Soil microbiome composition during *Morchella sextelata* cultivation

**Figure 2:**
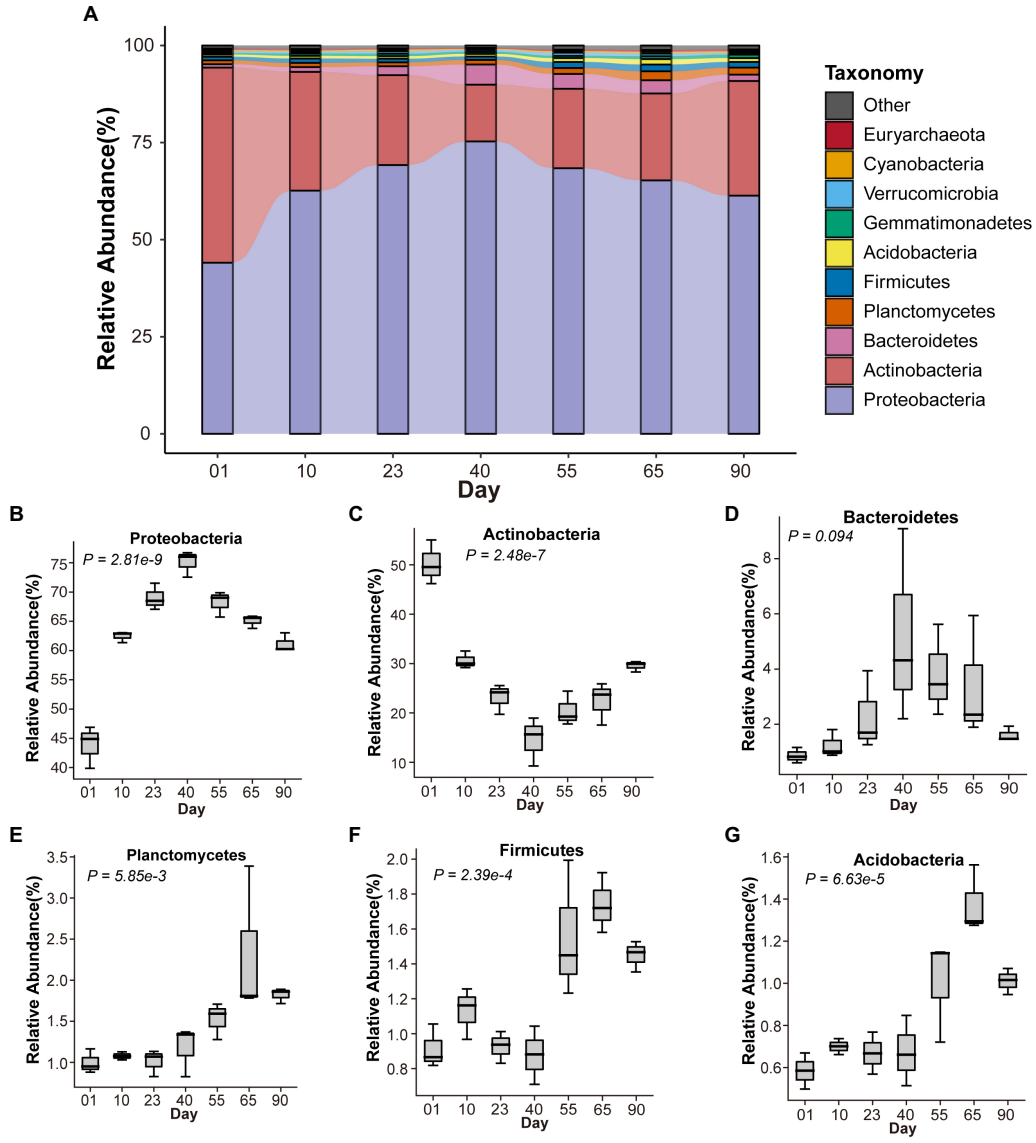
Composition of the soil microbiome at different growth stages of *Morchella sextelata*. **(A)** Relative abundances of the top 10 most abundant phyla at different time points. **(B–G)** Variation in microbial phyla during *M. sextelata* cultivation.

A total of 306.59 GB of clean sequence data (11.17–21.32 GB per sample) were obtained from 21 samples (Supplementary Table S2), from which 21 phyla, 56 classes, 129 orders, 263 families, 717 genera, and 2,307 species were identified (Supplementary Table S3). Rarefaction analysis indicated that the sequencing depth was sufficient to cover most microbial taxa (Supplementary Figure S1). The most dominant phyla were *Proteobacteria* and *Actinobacteria*, with a total abundance of 83.30%–95.09%. The abundances of other phyla were as follows: *Bacteroidetes* (0.61%–9.08%), *Planctomycetes* (0.83%–3.39%), *Firmicutes* (0.71%–1.99%), and *Acidobacteria* (0.50%–1.56%). As shown in Figure 2A, where the top 10 phyla with the highest relative abundances are displayed as a stacked bar plot, the relative abundance of different soil microbes fluctuated over time at different growth stages of *M. sextelata*; this was especially true at the primordium stage (day 40), an obvious turning point.

Phyla with a relative abundance > 1% but significantly differing over time are shown in Figure 2. The relative abundance of *Proteobacteria* continuously increased from mycelium (days 01, 10, and 23) to primordium (day 40) stages and decreased during the fruiting body stage (days 55, 65, and 90; *p* = 2.81 × 10^−9^, Figure 2B). The fluctuation in abundance of *Proteobacteria* mainly took place in the families *Burkholderiaceae*, *Comamonadaceae*, *Pseudomonadaceae*, *Sphingomonadaceae,* and *Xanthomonadaceae*. The abundance of *Actinobacteria* (*Nocardioidaceae*, *Pseudonocardiaceae,* and *Streptomycetaceae*) continuously decreased from mycelium (days 01, 10, and 23) to primordium (day 40) stages and increased during the fruiting body stage (days 55, 65, and 90; *p* = 2.47 × 10^−7^, Figure 2C). The relative abundance of *Bacteroidetes* was similar to *Proteobacteria* (*p* = 0.094, Figure 2D). *Planctomycetes* (*p* = 5.86 × 10^−3^, Figure 2E), *Firmicutes* (*p* = 2.39 × 10^−4^, Figure 2F), and *Acidobacteria* (*p* = 6.63 × 10^−5^, Figure 2G) had higher abundances during the fruiting body stage (days 55, 65, and 90) than at the other two stages.

### 3.2. Community diversity of the soil microbiome across the three growth stages of *Morchella sextelata*

**Figure 3:**
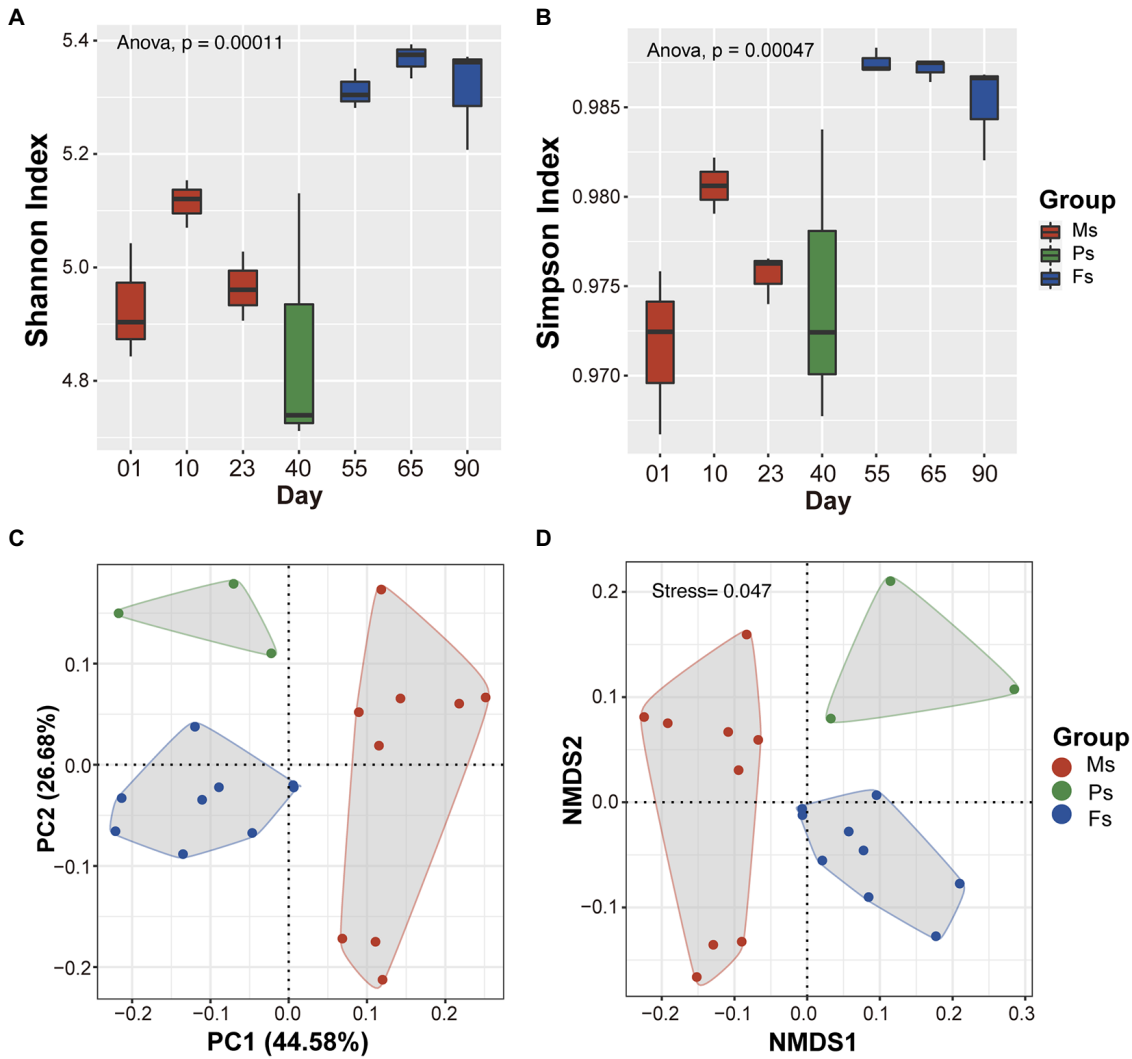
Variation in soil microbial diversity among *Morchella sextelata* growth stages. **(A,B)** Changes in microbial community alpha diversity based on Shannon’s and Simpson’s indexes. **(C,D)** PCoA and NMDS plots of microbial community diversity based on Bray–Curtis distances. Mycelium, primordium, and fruiting body stages are depicted in red, green, and blue, respectively. Ms, mycelium stage; Ps, primordium stage; Fs, fruiting body stage.

Soil microbial alpha diversity was significantly different among different growth stages (*p* < 0.01; Figures 3A,B). Shannon’s and Simpson’s index values were lowest at the primordium stage and highest at the fruiting body stage (Figures 3A,B). PCoA based on Bray–Curtis distances uncovered significant shifts in microbial community composition between stages (Figure 3C). In particular, soil microbial community compositions clustered into three groups closely correlated with mycelium, primordium, and fruiting body stages, which were separated along the main coordinate axis. Similar patterns were also observed in the NMDS analysis (Figure 3D). These results indicate that soil microbial community composition and structure varied during the different *M. sextelata* growth stages.

### 3.3. Key microbial biomarkers associated with *Morchella sextelata* growth stages

**Figure 4:**
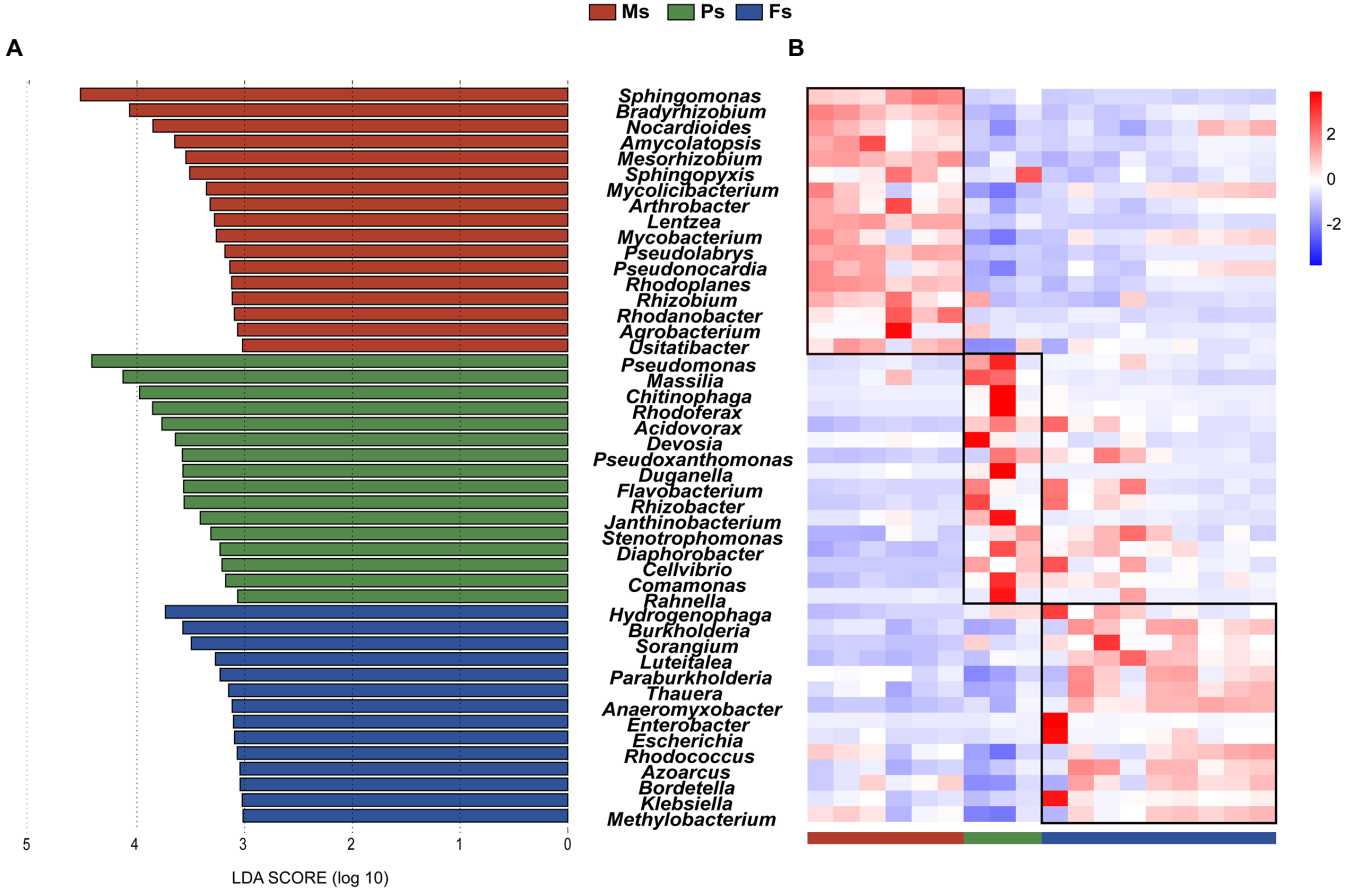
Identification of key microbial biomarkers associated with *Morchella sextelata* growth stages. **(A)** LEfSe to identify characteristic biomarkers between different stages. Only microbial taxa with a LDA score > 3 are shown. **(B)** Heatmap showing the relative abundances (*Z*-scores) of 47 stage-associated biomarkers identified by LEfSe. Ms, mycelium stage; Ps, primordium stage; Fs, fruiting body stage.

The LEFSe analysis was conducted to identify characteristic biomarkers associated with *M. sextelata* growth stages. Because the soil microbiome immediately after sowing mostly reflected the original status of the soil (before mycelial growth) and thus could have biased the stage-associated results, samples from day 01 were not included in the analysis. A total of 47 microbial biomarkers (LDA > 3), mainly from *Actinobacteria*, *Bacteroidetes,* and *Proteobacteria* were identified (Figure 4A). Two of 17 characteristic microbial taxa associated with the mycelium stage, namely *Sphingomonas* and *Bradyrhizobium*, had LDA values >4. We identified 16 microbial biomarkers associated with the primordium stage; among them, *Pseudomonas* and *Massilia* had the highest LDA values (>4). Finally, 14 microbial biomarkers were associated with the fruiting body stage, including *Hydrogenophaga* and *Burkholderia* with LDA values > 3. Each characteristic biomarker had its highest abundance at a specific *M. sextelata* growth stage (Figure 4B). As representative microbes of different stages, these biomarkers may extensively interact and play important roles in mycelium elongation, primordium formation, and fruiting body maturation to promote *M. sextelata* growth and development.

### 3.4. Validation of primordium-associated microbial biomarkers through comparison of soil samples with and without primordium formation

For use as controls, we collected five soil samples from a nearby greenhouse (distance < 500 m) in which *M. sextelata* had been sown at approximately the same time but with no primordium formation taking place (Supplementary Table S1). These soil samples had been managed and collected in the same way as the other samples in this study. To verify the characteristic biomarkers associated with the primordium stage, we horizontally compared the rhizosphere soil microbiome of the five control samples with that of samples with primordium formation.

**Figure 5:**
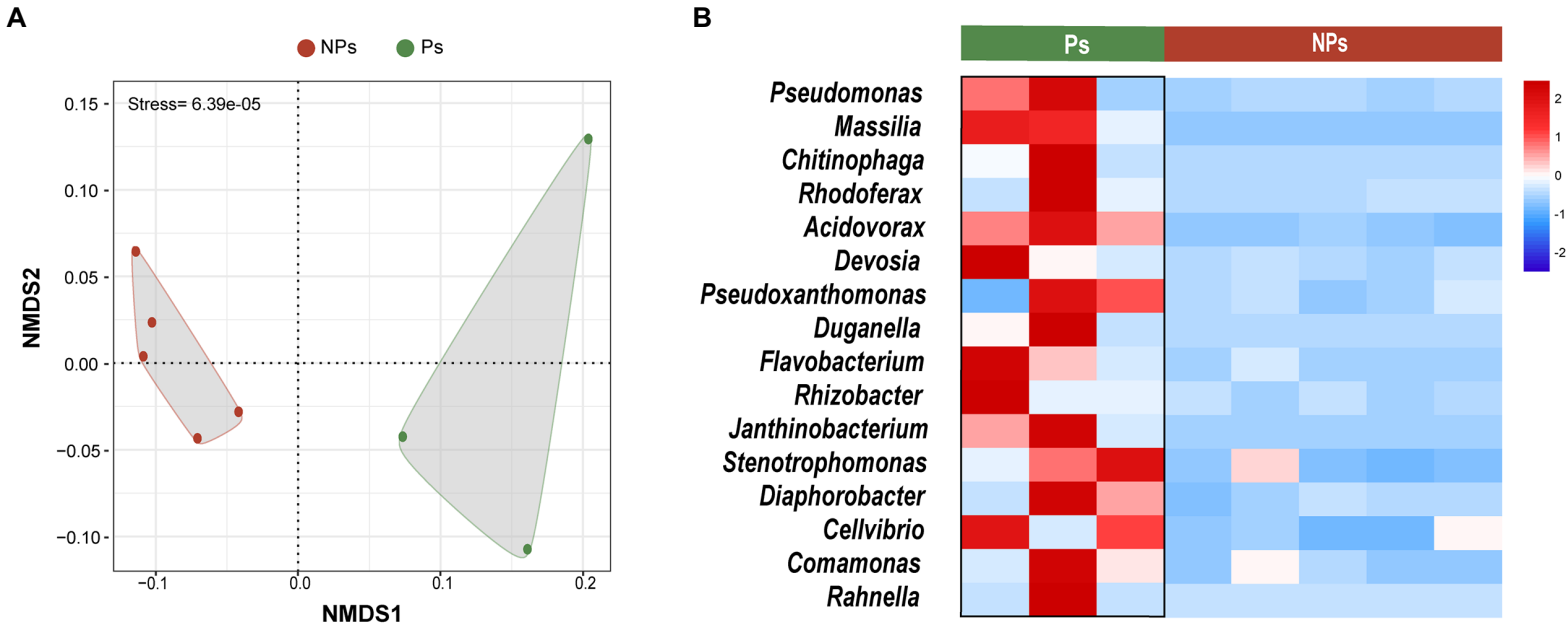
Comparative analysis of the soil microbiome with and without primordium formation. **(A)** NMDS plots of microbial community diversity based on Bray–Curtis distances. **(B)** Heatmap showing the relative abundances (*Z*-scores) of 16 primordium-associated biomarkers. Ps, samples with primordium formation (sampling at day 40). NPs, samples without primordium formation (sampling between days 55 and 65).

Alpha diversity was significantly different between the two sample groups (Shannon’s index, *p* = 0.0044; Simpson’s index, *p* = 0.024; Supplementary Figure S2). Microbial community structure differed significantly as well (Figure 5A). NMDS separated the samples into two independent groups, i.e., with and without primordium formation (Figure 5A). In addition, we predicted 16 primordium-associated biomarkers by LEFSe (Figure 4A) and further visualized their relative abundances with a heatmap (Figure 5B). Compared with their abundances in soil without primordium formation, all 16 biomarkers were significantly more abundant in soil with primordium formation. The absence of synergy among these microbes might be correlated with the failure of primordia to form.

### 3.5. Functional analysis of the soil microbiome across the three growth stages of *Morchella sextelata*

To explore biological functions of the soil microbiome during the life cycle of cultivated *M. sextelata*, we performed a functional annotation analysis at different *M. sextelata* growth stages based on the KEGG orthology database. The majority of enriched KEGG pathways were related to metabolism (55.47%), followed by environmental information processing (8.27%), genetic information processing (7.69%), human diseases (7.58%), cellular processes (7.20%), organismal systems (3.30%), and those not included in KEGG pathway or BRITE databases (10.49%; Supplementary Figure S3). Soil microbiome functional pathways are listed in Supplemental Table S4. Alpha diversity based on Shannon’s and Simpson’s indexes was significantly higher at the primordium stage than at mycelium and fruiting body stages (Supplementary Figure S4), thus indicating that the soil microbial community was functionally more diverse at the primordium stage. Principal component analysis (PCA) revealed that ecological functions of the soil microbiome varied among *M. sextelata* growth stages, with samples from the primordium stage found to be strikingly different from those of mycelium and fruiting body stages (Supplementary Figure S4).

**Figure 6:**
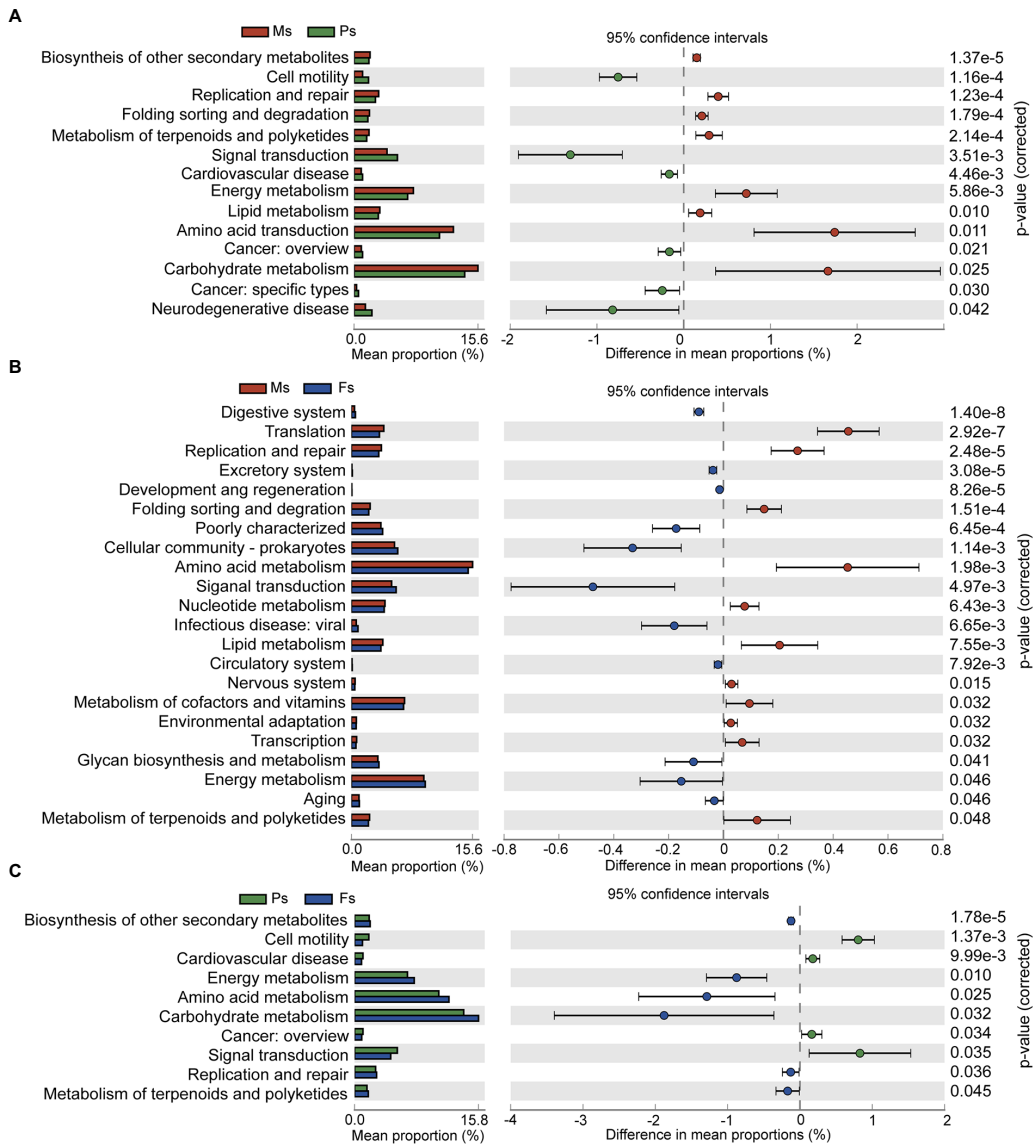
Variations in KEGG functions of the soil microbiome during mycelium, primordium, and fruiting body stages of *Morchella sextelata*. **(A–C)** Comparisons of mycelium and primordium stages **(A)**, mycelium and fruiting body stages **(B)**, and primordium and fruiting body stages **(C)**. Ms, mycelium stage; Ps, primordium stage; Fs, fruiting body stage.

Pairwise comparative analysis of level-2 KEGG pathways was used to characterize the functional pathways differing among the three *M. sextelata* growth stages (Figure 6). Pathways related to carbohydrate, amino acid, and energy metabolism were enriched at the mycelium stage (Figures 6A,B), an observation supporting the idea that the soil microbiome helps *M. sextelata* mycelium absorb and transform large-scale nutrients from surrounding substrate. At the primordium stage, the soil microbiome was enriched in signal transduction-related pathways (Figures 6A,C), including those associated with the mitogen-activated protein kinase signaling pathway and the bacterial two-component regulatory system. This result suggests that soil microbes can transmit information through signal transduction molecules to transform rhizosphere microbes during primordium formation, a critical step in morel cultivation. At the fruiting body stage, ascocarp development and maturity were accompanied by a significant increase in enriched pathways related to carbohydrate, amino acid, and energy metabolism, thus reflecting potential nutrient demand during morel fruiting.

## 4. Discussion

### 4.1. Microbial community dynamics across three *Morchella sextelata* growth stages

The main components of the soil microbiome during the three growth stages of *M. sextelata* were *Proteobacteria* and *Actinobacteria*, followed by *Bacteroidetes*, *Planctomycetes*, *Firmicutes*, and *Acidobacteria* (Figure 2A). These phyla are also the major constituents of the soil microbial community in the natural habitat of *M. rufobrunnea* (Orlofsky et al., 2021) and in semi-synthetic substrates of *M. importuna* (Tan et al., 2021), which indicates that these microbes are indispensable during the morel life cycle. Nevertheless, the composition of the soil microbiome was marked by significant differences among mycelium, primordium, and fruiting body stages (Figures 3C,D). In particular, the relative abundances of *Proteobacteria* and *Bacteroidetes* increased until the primordium stage and then decreased, whereas the opposite was true of *Actinobacteria* (Figures 2B,C). Similar patterns have been observed in studies of rhizosphere microbiomes of *A. bisporus* (Kertesz and Thai, 2018), *G. lucidum* (Zhang et al., 2018), and *P. portentosus* (Yang et al., 2019), with *Proteobacteria* more abundant at the primordium stage than at other growth stages. The fact that microbiome structure varied significantly among growth stages strongly suggests that morels can shape the soil microbiome and that normal morel growth requires specific microbes. These findings may be useful for the future improvement of morel cultivation through microbial inoculants.

### 4.2. Keystone microbes and their functions during *Morchella sextelata* growth

To further explore microbiota having critical effects on the growth of *M. sextelata*, we identified 47 characteristic biomarkers associated with specific growth stages (Figure 4A). Among them, *Sphingomonas* and *Bradyrhizobium* were significantly abundant at the mycelium stage and thus might contribute to the mycelium elongation and nutrient metabolism of morels. Interestingly, *Sphingomonas* and *Bradyrhizobium* are considered to be beneficial to the growth of mushrooms, which can multiply during composting and have strong lignocellulose degradation ability (Vajna et al., 2012; Zhang et al., 2014; Gohar et al., 2022). In this way, the surrounding substrate can be degraded to release more nutrients for mushroom growth.

At the primordium stage, *Pseudomonas* was the main biomarker (Figure 4A). *Pseudomonas* promotes mycelium growth, primordium formation, and high yield in *A. bisporus* (Noble et al., 2009; Zarenejad et al., 2012; Chen et al., 2013), *Pleurotus eryngii* (Kim et al., 2008), and *Pleurotus ostreatus* (Cho et al., 2003), which suggests that *Pseudomonas* can also interact directly with morels to induce primordium formation. This hypothesis is supported by the fact that *Pseudomonas* was significantly more abundant in our study in soil with primordium formation than in soil with failed primordium formation (Figures 5A,B). *Burkholderiaceae* (*Hydrogenophaga* and *Burkholderia*) was present in higher abundance in the fruiting body stage (Figure 4A). Members of *Burkholderiaceae* have been associated with the formation of *P. portentosus* (Yang et al., 2019) and *Tricholoma matsutake* (Kataoka et al., 2012; Li et al., 2016), which suggests that this phylum promotes the growth of morel ascocarps. Given the important functions of these keystone microbes in morel growth and development, the use of inoculants containing such microbes may be a practical way to promote mycelium elongation, primordium formation, and ascocarp maturation in morels.

### 4.3. Potential pathogenic microbes during *Morchella sextelata* cultivation

Soil-borne pathogenic fungi and bacteria have been reported in morel cultivation (Guo et al., 2016; He et al., 2017, 2018). We thus note that some members of *Pseudomonas*, aside from their benefits, have ecologically harmful effects and can cause mushroom diseases. For example, mushroom brown spot disease due to *Pseudomonas tolaasii* has caused yield losses in *A. bisporus* (Largeteau and Savoie, 2010), *Pleurotus ostreatus* (Lo Cantore and Iacobellis, 2014), and *Flammulina velutipes* (Han et al., 2012). In our study, *Pseudomonas* was significantly enriched at the primordium stage (Figure 4B) and was more abundant in soil with primordium formation (Figure 5B). This result suggests that this genus plays an important role in promoting primordium formation. The application of growth-promoting bacteria as microbial inoculants is a promising method (Nerva et al., 2022; Upadhyay et al., 2022; Upadhyay and Chauhan, 2022). Consequently, the addition of specific beneficial microbial taxa to promote growth and enhance the resistance of *M. sextelata* is worthy of further investigation.

In conclusion, our study has revealed the dynamics of the soil microbiome throughout the *M. sextelata* cultivation life cycle. We uncovered significant changes in the microbial community during *M. sextelata* growth and identified stage-associated biomarkers. Our functional analysis of the soil microbiome revealed that diverse functional strategies are required for different growth stages of *M. sextelata*. The presented data provide novel insights for future application of microbial inoculants and soil improvement for stable morel cultivation.

## Data availability statement

The data presented in the study are deposited in the NCBI repository (https://www.ncbi.nlm.nih.gov/), accession number PRJNA841746.

## Author contributions

CZ, XS, and WW conceived and designed the research. CZ, JZ, and YZ coordinated the sampling. CZ and XS processed, analyzed, and interpreted the data and wrote the original draft. All authors contributed to the article and approved the submitted version.

## Funding

This work was supported by the National Key Research and Development Program of China (2021YFD1600404) and the Postdoctoral Directional Training Foundation of Yunnan Province.

## Conflict of interest

YZ was employed by the company Shandong Junsheng Biotechnologies Co., Ltd.

The remaining authors declare that the research was conducted in the absence of any commercial or financial relationships that could be construed as a potential conflict of interest.

## Publisher’s note

All claims expressed in this article are solely those of the authors and do not necessarily represent those of their affiliated organizations, or those of the publisher, the editors and the reviewers. Any product that may be evaluated in this article, or claim that may be made by its manufacturer, is not guaranteed or endorsed by the publisher.
