# Supplementary material for: Dynamics of soil microbiome throughout the cultivation life cycle of morel (Morchella sextelata)
Source: Front Microbiol. 2023 Feb 22;14:979835. doi: 10.3389/fmicb.2023.979835 (PMC9992412; doi:10.3389/fmicb.2023.979835)
Supplement: Supplementary file 1 [file Data_Sheet_1.DOCX]

Supplementary Material

**
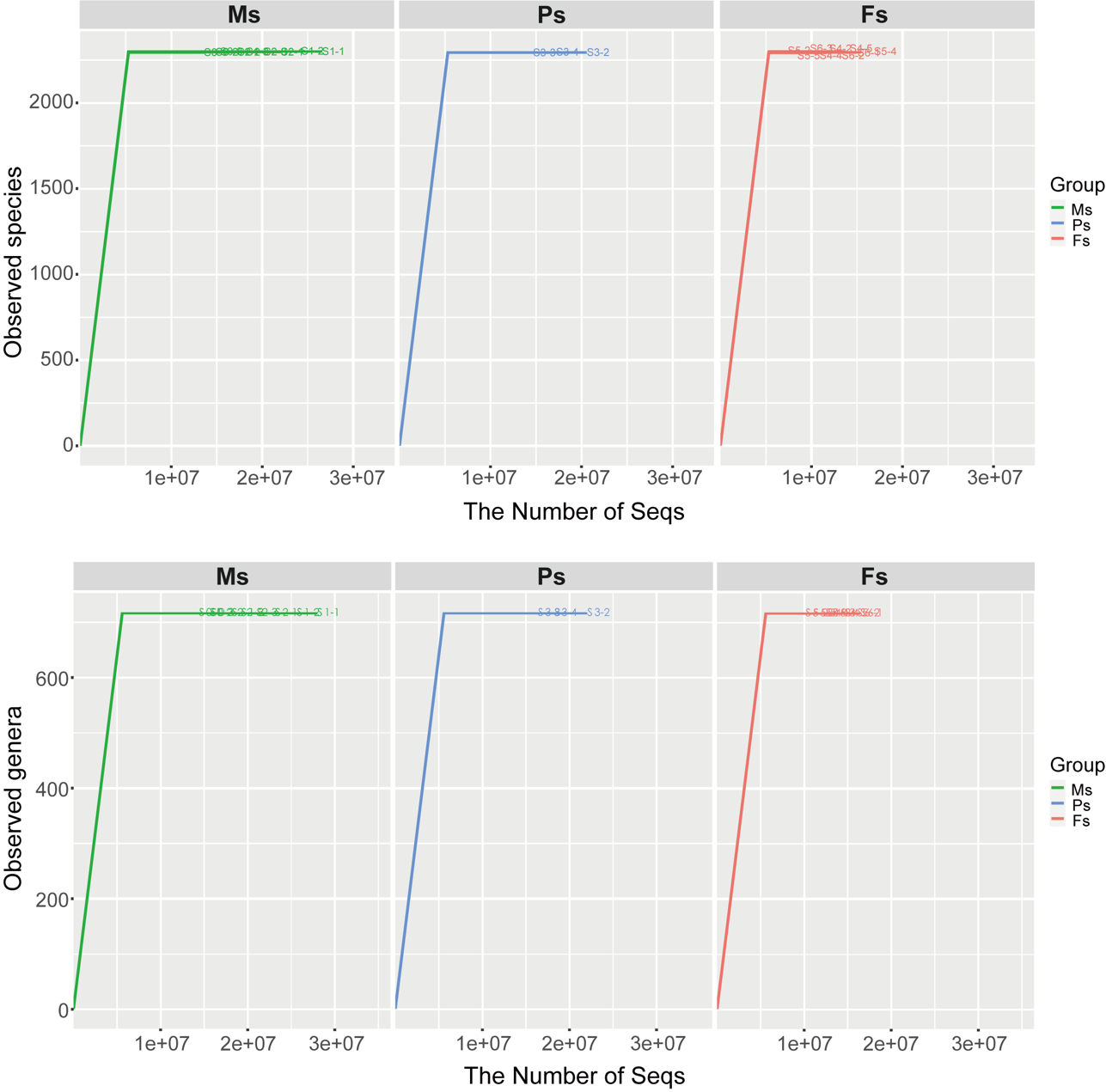
**

**Supplementary Figure S1.** The rarefaction curves of observed species and genera among all samples. Ms, mycelium stage; Ps, primordium stage; Fs, fruiting body stage.

**
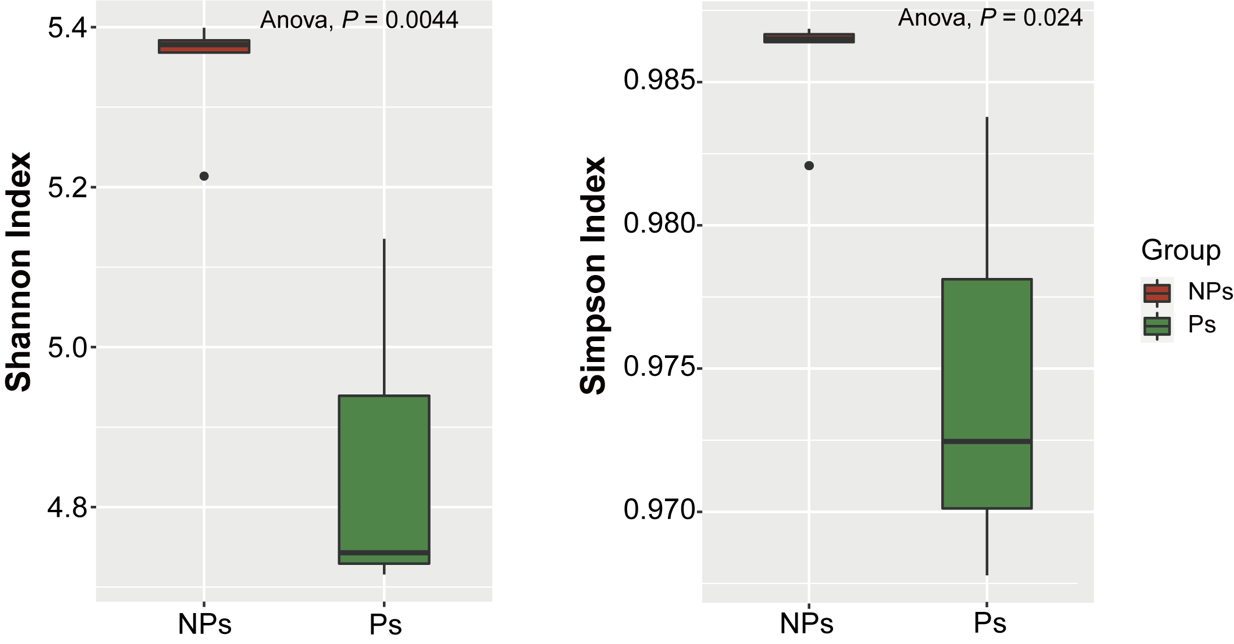
**

**Supplementary Figure S2.** The Alpha diversity of soil microbiome with and without primordium formation. Ps, samples with primordium formation. NPs, samples without primordium formation. The Ps and NPs are depicted in green and red respectively.

**
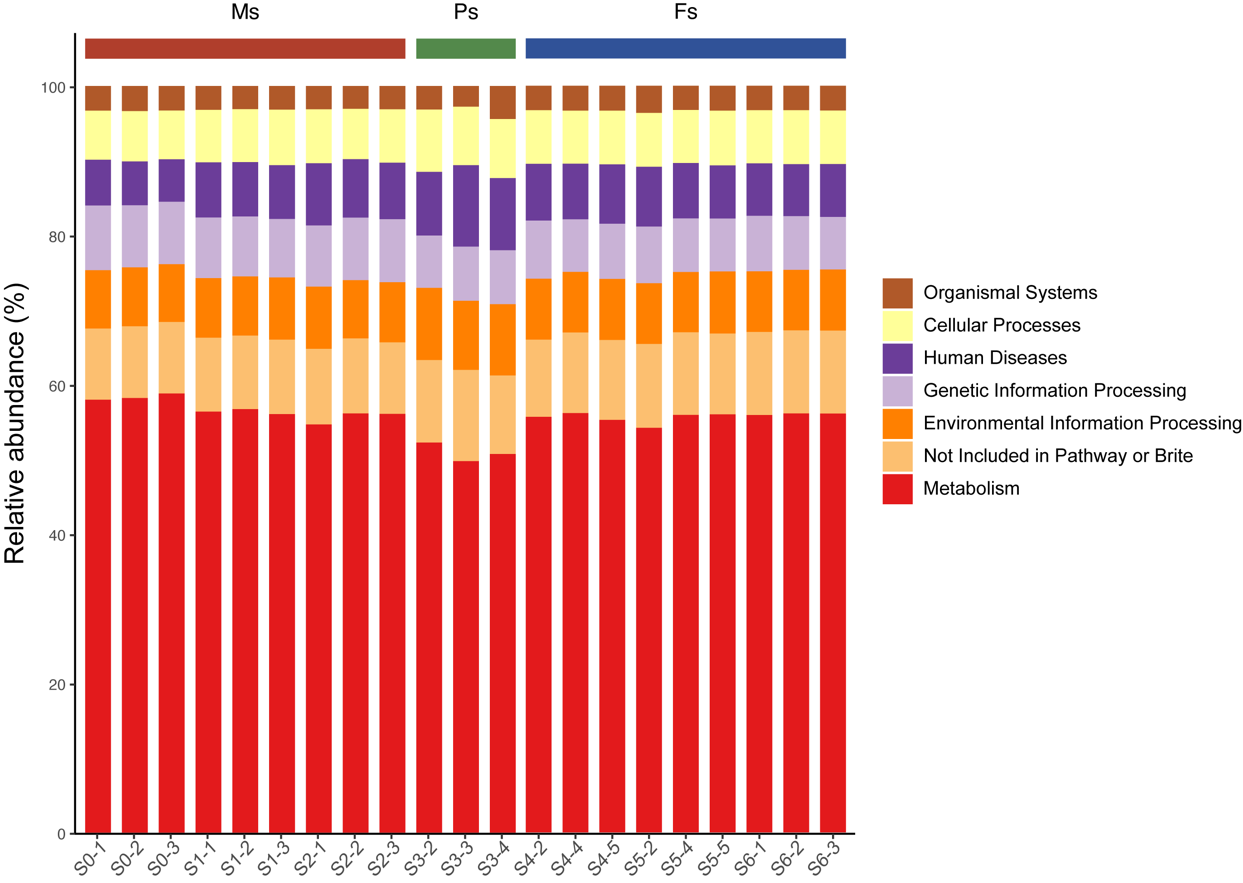
**

**Supplementary Figure S3.** The relative abundance of Level-1 KEGG functional pathways in the three *Morchella sextelata* growth stages. Ms, mycelium stage. Ps, primordium stage. Fs, fruiting body stage.

**
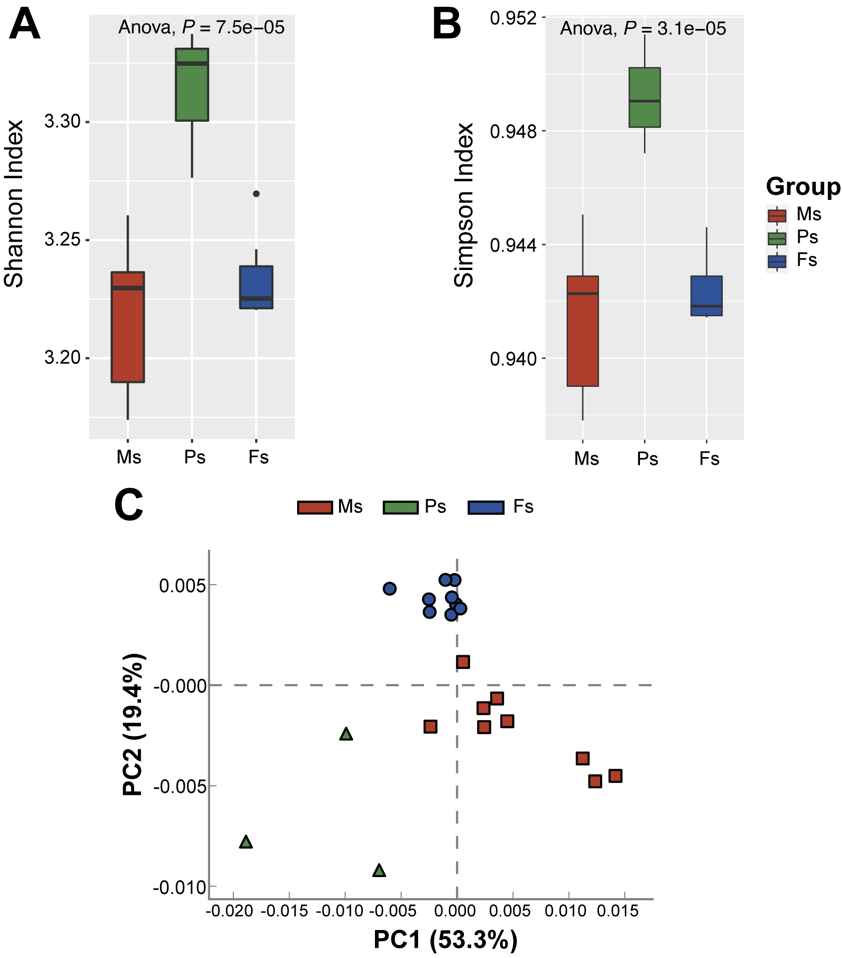
**

**Supplementary Figure S4.** Alpha and beta diversity of the soil microbiome functional pathways in the three *Morchella sextelata* growth stages. (A-B) α-diversity boxplots of microbial functional pathways based on the Shannon and Simpson index. (C) Principal components analysis (PCA) of the function al pathways of soil microbiome. Ms, mycelium stage. Ps, primordium stage. Fs, fruiting body stage.

**Supplementary Table S1.** The detailed sample information in this study (see separate excel file).

**Supplementary Table S2.** The sequencing statistics for all soil samples in this study (see separate excel file).

**Supplementary Table S3.** The microbial taxonomic tables of soil microbiome in the three *Morchella sextelata* growth stages (see separate excel file).

**Supplementary Table S4.** The functional composition tables of soil microbiome in the three *Morchella sextelata* growth stages (see separate excel file).
